# A prospective study of maternal preference for indomethacin prophylaxis versus symptomatic treatment of a patent ductus arteriosus in preterm infants

**DOI:** 10.1186/s12887-015-0353-4

**Published:** 2015-04-22

**Authors:** Khalid AlFaleh, Eman Alluwaimi, Ahlam AlOsaimi, Sheikha Alrajebah, Bashayer AlOtaibi, Fatima AlRasheed, Turki AlKharfi, Bosco Paes

**Affiliations:** Department of Pediatrics (Division of Neonatology), College of Medicine, King Saud University, Riyadh, Saudi Arabia; Department of Pediatrics, King Fahad Medical City, Riyadh, Saudi Arabia; Department of Pediatrics (Neonatal Division), McMaster University, Hamilton, ON Canada

**Keywords:** Indomethacin prophylaxis, Patent ductus arteriosus treatment, Decision aid, Preterm, Maternal preference

## Abstract

**Background:**

The management of a patent ductus arteriosus in preterm infants continues to be debated among neonatologists due to the absence of concrete evidence that precisely weighs the long term outcomes of active, early intervention against a conservative approach. In the majority of institutions, parents are encouraged to play an active role in the complex, decision –making processes with regard to the care of their infants. The objective of this study is to elicit maternal preferences for indomethacin prophylaxis versus treatment of a patent ductus arteriosus (PDA) in extremely low birth weight (ELBW) infants, utilizing a decision aid instrument (DAI).

**Methods:**

Healthy and high risk pregnant women at 23–28 weeks gestation, and mothers of admitted ELBW infants were enrolled. A computer based, validated DAI was utilized during interviews. The DAI first provides information about prematurity and concurrent morbidities with comprehensive facts of the pros and cons about prophylactic versus treatment options. It subsequently coaches participants how to select values and preferences based on their decisions. A 17-item questionnaire assessed and valued each short and long term morbidity of extreme prematurity and preferred choice for PDA management.

**Results:**

Two hundred ninety nine subjects were enrolled; 75% were healthy women at 23–28 weeks gestation, 19% were high risk and 6% recently delivered an ELBW infant. Eighty-two percent preferred a prophylactic indomethacin strategy versus symptomatic treatment for the management of PDA. Across a spectrum of potential morbidities, the occurrence of severe intraventricular hemorrhage was viewed by mothers as the most un-wanted outcome irrespective of the two proposed options.

**Conclusions:**

In contrast to neonatal practitioners, mothers who used this particular DAI strongly endorsed prophylactic indomethacin versus a treatment intervention for the management of PDA in preterm infants.

**Electronic supplementary material:**

The online version of this article (doi:10.1186/s12887-015-0353-4) contains supplementary material, which is available to authorized users.

## Background

The finding of a patent ductus arteriosus (PDA) is very common among very low birth weight infants. The delay in closure of the ductus is inversely related to gestational age varying from 20% in premature infants greater than 32 weeks, up to 60% in extremely low birth weight infants (ELBW; < 1000 g) [1,2]. Persistence of a PDA may result in a significant left to right shunt with an increase in left ventricular output. Although the duct usually closes spontaneously by five days of age in most infants > 30 weeks’ gestational age, it remains patent over the same duration in more than two thirds of infants who are < 30 weeks [3]. In preterm infants, a hemodynamically significant PDA is associated with many important short and long term morbidities including necrotizing enterocolitis, bronchopulmonary dysplasia and intraventricular hemorrhage (IVH) [4-7]. Significant shunting through the ductus may result in lower superior venacaval flow and subsequent occurrence of late IVH [8]. Lastly, large left to right ductal shunting is associated with a significant increase in pulmonary blood flow and serious pulmonary hemorrhage [9,10]. The management of a PDA remains one of the most controversial issues in neonatal care despite the extensive body of scientific literature addressing either prevention or treatment, since the goal of the chosen strategy is to primarily reduce harm and influence positive outcomes in the preterm host.

Although prophylactic indomethacin is proven to decrease the incidence of symptomatic PDA (50%), surgical PDA ligation (50%), IVH (35%), and pulmonary hemorrhage [11,12], clinicians remain uncertain whether to utilize this intervention or treat a symptomatic PDA because of the lack of significant improvement in neurosensory outcomes [13,14] and chronic lung disease [15,16] in infants receiving indomethacin prophylaxis. Indomethacin use is also associated with numerous side effects including increased risk of renal impairment [17,18], fluid retention, necrotizing enterocolitis [19] and potential alterations in cerebral blood flow velocity [20], coronary artery perfusion [21], and regional tissue oxygenation [22].

Recently, clinical practice has expanded from traditional authoritative models, in which physicians make treatment decisions for patients, to include shared decision-making. This involves an exchange of information to prepare patients to make treatment decisions and engage in the process of decision-making with their healthcare providers [23-25]. We have recently developed and validated a computerized, interactive, structured decision aid instrument (DAI) to elicit parents’ preferences with regard to indomethacin prophylaxis versus symptomatic treatment in the management of their premature infants [26]. The DAI was shown to significantly improve both knowledge and decisional conflict based on a 5-point Likert scale [26].

The primary objective of this study was to elicit maternal preferences with regard to indomethacin prophylaxis versus symptomatic treatment in the management of their preterm infants, utilizing a validated, computerized DAI.

## Methods

We conducted a prospective structured survey from October 2011 to March 2012 in three tertiary, perinatal centers in Riyadh, Saudi Arabia (King Khalid University Hospital, King Fahad Medical City and Dr Sulaiman AlHabib Medical Group). The study was approved by the Institutional Ethics Board at the College of Medicine, King Saud University.

Our survey included healthy pregnant women with a gestational age between 23–28 weeks who were identified in the antenatal clinics, high risk pregnant women with a similar gestational age hospitalized in the antenatal wards, and mothers of ELBW infants admitted to the neonatal intensive care unit. The eligibility of pregnant patients was decided after reviewing the medical records for accurate documentation of maternal gestational age based on a precise last menstrual period or first trimester ultrasound and whether an underlying medical condition placed a woman in the high risk category for preterm delivery. Potential subjects were asked to participate in the study. After obtaining a signed consent, enrolled subjects were interviewed by a team member in the prenatal outpatient clinics, antenatal wards or neonatal intensive care unit. Participants were asked to browse through and read the DAI displayed on a laptop and were supervised by a member of the research team. Identified queries were addressed and uncertainties resolved. Enrollees were then asked to answer a 17-item questionnaire during which they assessed and valued the short and long term morbidity of extreme prematurity and subsequently integrated the information provided to make a decision on whether to use prophylactic indomethacin or treatment of a symptomatic PDA. Adequate time was permitted to facilitate the process in order to ensure that the subjects selected a preferred intervention based fully on the knowledge acquired through the DAI.

The DAI as previously described (Additional file 1) [26], is structured in Arabic and has two components. The first part provides medical information and guided instruction on how to use the DAI, facts on prematurity and its potential complications, and knowledge about the use of indomethacin for PDA in ELBW infants based on two options, namely prophylactic therapy and symptomatic treatment of a PDA. This section also comprises data for both options on the chance of short term consequences (risks relative to PDA, surgical PDA ligation and severe IVH) and the chance of long term outcomes (survival and neurosensory impairment), and potential adverse effects (gastrointestinal perforation and renal impairment) specifically related to indomethacin prophylaxis. Through a series of screen displays, well balanced information is presented systematically in simplified language, such that it is easy for the subject to comprehend. Pertinent material on each topic is outlined in a format that includes both paragraphs and applicable bullet points. In addition, parents were presented with pictures depicting cerebral palsy in children in order to visually grasp the seriousness of the neurosensory outcomes. Explanations of other potential deficits such as deafness, blindness and cognitive impairment were verbally communicated by the site investigators. Therefore the severity of neurodevelopmental compromise was fully portrayed through the DAI for the parents and a selection bias for prophylaxis was minimized.

In the second part; the DAI trains participants on the decision making process regarding the use of indomethacin for the management of a PDA in preterm infants. Several case scenarios of a PDA are delineated with a succinct description of the side effects of indomethacin, clarification of the patient’s own values for each therapeutic benefit and harm, while affording assistance with final decision making. A verbal description of uncertainty is utilized (e.g. likely) rather than a numeric one (e.g. 0.60) in order to streamline the assignment while lending credence to the established fact that verbal communication is as effective as an allocated numeric entity [24]. However, the frequencies of outcomes are also displayed on the DAI [26].

Participants are then asked to assign a value for each potential outcome associated with indomethacin therapy. The DAI presents the values as a horizontal scale ranging from 0 (worst outcome i.e. death) to 100 (optimum health condition) in increments of 1 unit. A cursor on the scale is utilized to assign a personal value for a specific outcome which appears in the box adjacent to the scale. The computer derived information is collectively synthesized and participants are then permitted to make one of two possible choices based on data entry, “to use prophylactic indomethacin” or elect for “symptomatic treatment with indomethacin”. The team was informed a priori neither to intervene with the selected maternal option nor to impart their opinion with regard to ideal outcomes. Participants could electively abandon the DAI at any stage and terminate the study.

The primary outcome was the percentage of women who preferred either indomethacin prophylaxis or symptomatic treatment for the management of a PDA. Additional data were collected by self-written surveys completed by eligible mothers immediately after concluding the task on the DAI. The surveys included the following baseline variables: education level, job status, computer or internet user or not and maternal gestational age at the time of the interview (Tables 1 and 2).

**Table 1:** **Baseline characteristics of interviewed women**

**Variable**	**No. (%)**
**Education**	
Primary	17 (5.7%)
Intermediate	40 (13.4%)
High School	117 (39.1%)
College	115 (38.5%)
Master’s Degree	7 (2.3%)
PhD	3 (1%)
**Occupation**	
Housewife	184 (61.5%)
Employed	115 (38.5%)
**Computer user**	
No	32 (10.7%)
Yes	267 (89.3%)
**Internet user**	
No	36 (12%)
Yes	263 (88%)
**Type of pregnancy**	
Normal	222 (74.5%)
High Risk	57 (19.1%)
Recently delivered an extremely low birth weight infant	19 (6.4%)
**History of previous prematurity**	
No	257 (86%)
Yes	42 (14%)
**Gestational month at time of interview**	
5	20 (7%)
6	166 (58%)
7	101 (35%)

**Table 2:** **Logistic regression of baseline variables and maternal choice of indomethacin therapy**

**Baseline Variable**	**OR**	**95% CI**	**p-value**
Age	0.98	0.93, 1.02	0.33
Education	1.09	0.79, 1.49	0.6
Occupation	2.2	1.1, 4.27	0.02
Computer user	1.12	0.43, 2.89	0.82
Internet user	1.43	0.61, 3.35	0.41
Number of pregnancies	0.98	0.87, 1.11	0.81
Number of children	0.97	0.84, 1.12	0.69
Current gestational age	0.65	0.39, 1.08	0.09
Type of pregnancy	0.97	0.58, 1.6	0.89
History of previous prematurity	1.62	0.6, 4.35	0.33

Omnibus Likelihood Ratio between maternal characteristics and final maternal choice (chi-square [df], p-value); 11.8 [10], 0.29).

### Sample size and analysis

We planned a convenience sample of 300 mothers. Mothers who had not previously experienced a preterm birth were included as part of the cohort since they comprise an unbiased group. Data were analyzed as count and percentages for categorical variables and mean and standard deviation for continuous variables using the statistical software program SAS version 9.3. To evaluate a significant association between maternal treatment choice and the baseline variables, a multiple logistic regression analysis model was utilized. Sub-analyses were conducted of the preferred option for the management of a PDA versus the maternal values placed on short and long term outcomes. A p-value < 0.05 was considered statistically significant.

## Results

Two hundred and ninety nine eligible women were included. None elected to withdraw after starting the interview. Of these; 75% were healthy, pregnant women at 23 to 28 weeks gestation who were recruited from the antenatal clinics, 19% were of similar gestational age and considered high risk and were enrolled on the antenatal wards, and 6% had recently delivered an ELBW infant. Most interviewed participants had a high school or more advanced educational level, were unemployed and had no previous history of preterm birth (Table 1).

For our primary outcome, 82% of enrolled mothers preferred a prophylactic indomethacin strategy for their expected or delivered preterm infant. On multivariable logistic regression, there was no significant correlation between maternal characteristics and final maternal choice (Omnibus Likelihood Ratio; p = 0.29) (Table 2). Most short term neonatal outcomes of prematurity such as a PDA, PDA ligation, severe IVH, and bronchopulmonary dysplasia were valued negatively by interviewed participants. The occurrence of severe IVH was the most unwanted outcome with a reported value of 29 (Range: 0–100; Figure 1). When maternal values were compared based on the therapeutic option chosen, women who chose the prophylactic approach significantly rated severe IVH as the worst outcome. On the other hand, women who preferentially selected symptomatic treatment of a PDA over prophylaxis rated oliguria significantly worse (Table 3). Neurosensory outcomes did not appear to influence the choice of treatment modality.

**Figure 1:**
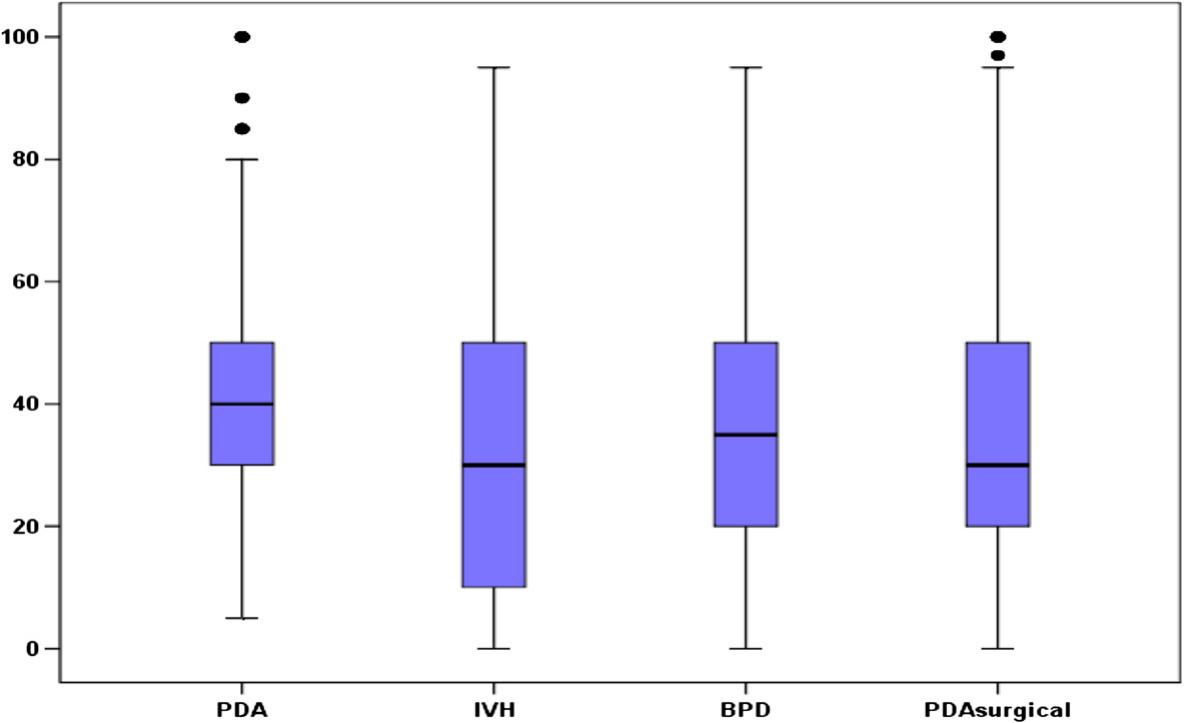
Distribution of maternal values regarding outcomes of extremely low birth weight infants. Results are shown as mean (standard deviation). BPD, bronchopulmonary dysplasia; IVH, intraventricular hemorrhage; PDA, patent ductus arteriosus.

**Table 3:** **Maternal values of neonatal outcomes favoring different therapeutic indomethacin options**

**Outcome**	**Women favored prophylactic approach Mean (SD)**	**Women favored symptomatic approach Mean (SD)**	**Mean difference (95% CI)**	**p- value**
PDA	41 (21)	44 (24)	−3 (−9, 3)	0.3
IVH	28 (23)	35 (25)	−7 (−14, −0,3)	0.04
BPD	35 (22)	40 (23)	−5 (−12, 1)	0.11
PDA ligation	38 (24)	39 (30)	−1 (−9, 6)	0.7
Oliguria	63 (22)	55 (21)	8 (2, 15)	0.01
Neurosensory outcome	65 (27)	58 (24)	7 (−1,14)	0.09

BPD, bronchopulmonary dysplasia; IVH, intraventricular hemorrhage; PDA, patent ductus arteriosus.

## Discussion

In the era of evidence based medicine, neonatal practitioners should always evaluate therapies or interventions for preterm infants within three domains; clinical experience, research evidence and parental preferences. In neonatal medicine and based on research evidence alone, the utilization of indomethacin prophylaxis as a strategy in the management of a PDA in ELBW infants declined dramatically after the publication of the Trial of Indomethacin Prophylaxis in Preterm (TIPP) infants study in 2001[27] owing to the lack of long term benefits. A recent paper by Clyman et al. [14], also reported that the use of indomethacin prophylaxis in the National Institute of Child Health and Human Development network increased after the publication of the Ment et al. trials in 1994 [28,29] but declined after the TIPP trial [27]. Neonatologists differ widely in their approach to PDA management [30], but in the majority the general trend now favors the use of indomethacin for a symptomatic, moderate to large PDA that is hemodynamically significant rather than prophylaxis [14,31,32].

In this study, we attempted to consolidate the third domain of evidence based medicine i.e. parenteral preferences with regard to indomethacin prophylaxis utilization for the management of a PDA in ELBW infants. The DAI is the first of its kind in the neonatal cardiology field. It followed a rigorous well defined developmental process, and provides user-friendly, accessibility and convenience to users [26]. The majority of expectant, healthy and high risk pregnant women or mothers of ELBW infants interviewed, preferred the prophylaxis approach in contrast to the widely adopted, physician-directed approach of only offering indomethacin therapy as a symptomatic treatment for a hemodynamically significant PDA. Participants in our study documented their perceived values of neonatal outcomes and reported severe IVH as the worst outcome that could affect their preterm infant. Parents who favored prophylaxis rated severe IVH worse and oliguria better than the treatment group. This serves to suggest that the study subjects did understand the content of the DAI and made decisions accordingly. It is therefore a testament to the success of the DAI in ‘teaching’ the study subjects, and provides face validity for the DAI.

There are two models in determining what defines the best interest of the preterm infant. First is the expert model of the neonatologist who is emotionally unattached to the infant but has the necessary knowledge regarding prognosis, makes decisions that are objective and are in the best interests of the infant from a healthcare provider perspective. The alternate model is the negotiated model that consists of a shared decision process where the physician using his expertise collaboratively with a family-centered approach based on maternal perceived values, garnered through the utilization of a well-structured DAI, guides the best decision for the infant. The negotiated model is superior and reduces decisional conflict and allows parents of ELBW infants to implement a strategy in the management of their infant based on their specific values [33].

The management of a PDA in preterm infants <28 weeks remains undoubtedly controversial in the neonatal arena [34-36]. The unanswered question is whether parents should be invited to participate in the decision making processes about PDA management of their infants when healthcare providers remain undecided on the optimal, evidence-based approach to the problem relative to the existing, published clinical trials. Parents play a pivotal role in critical decisions with regard to the best interests of their child. Such shared decisions traverse the boundaries of informed consent for investigative research [37-39], management of anticipated preterm birth at the threshold of viability [40,41], treatment of life-threatening conditions [42,43] inclusive of numerous practices and interventions in neonatal intensive care [44,45]. Bailey et al. [44] reported in a US survey of neonatologists, that 19%-41% of their decisions regarding the use of prophylactic indomethacin, the number of indomethacin courses for the symptomatic treatment of a PDA and PDA ligation, was influenced by parental conversations. In general family-centered care and parental decision making authority are held in high regard by healthcare professionals in the neonatal field with the proviso that ultimate, well-informed decisions should be made in the best interests of the child. The use of a DAI fosters better education of parents with regard to the management of a PDA in extremely premature infants and provides the first step of facilitating parental autonomy and engaging parents opinions and rights as part of the complex decision making process on the issue. It is important to recognize that the DAI is not offered as a replacement for honest and transparent discussions with parents about the pros and cons of the various approaches to management but rather as an attempt to deepen their understanding of the risks versus harm of the proposed strategies and to provide informed, shared decisions which may impact their infants’ treatment [46]. The DAI can be therefore adapted and specific elements modified to incorporate additional treatment options such as the conservative approach for the management of a PDA or a cardiac ultrasound-targeted intervention based on individualized neonatal intensive care practices.

There are several limitations that merit consideration with regard to our study. First, the majority of our sample included healthy or at risk pregnant women who did not experience having an extremely preterm infant, which may have influenced their decision, despite the information incorporated in the DAI. Second, a more valid decision making process would be expected if both parents were involved, however this was not possible as it is the norm in our culture to have pregnant mothers attend antenatal clinics alone. Moreover, although some institution review boards stipulate that consent should be sought from both parents, this requirement is not easily accomplished. In a large-scale population-based study almost 20% of fathers were not immediately available when mothers were approached for consent [47]. Third, irrespective of the selected approach to the management of a PDA, women did not perceive that neurosensory compromise was a severe morbidity based on their ratings. This may either be due to a lack of appreciation of the long term impact on a newborn or that parents and healthcare professionals truly view health-related quality of life states differently [48,49]. Saigal et al. [50] indicate that not only are there differences in the perceptions of health, but the appraisal of these perceptions are significantly different between children, parents and health professionals. The optimum management of a PDA remains controversial among neonatologists because of the lack of solid evidence supporting one, specific modality of treatment over another [51,52]. Hence, it is not surprising that parents may differ from neonatologists regarding what they perceive as the best treatment strategy for a PDA. Fourth, our interviewed participants were all of Saudi ethnicity and the information in the DAI is outlined in Arabic language which limits the generalizability of our results. Last, the DAI may have inadequately described neurodevelopmental impairment and this may have swayed parental decisions in favor of short term outcomes. However, the DAI does provide clear, detailed information about prematurity and outcomes expected with real life scenarios and mothers were additionally shown pictorial representations of cerebral palsy which were accompanied by verbal descriptions of blindness, auditory deficits and cognitive impairment, thereby facilitating an informed decision.

We do believe that our study will set the stage to revisit the issue of indomethacin therapy factoring in parental wishes and values into the decision making process either immediately after an infant is admitted to the neonatal intensive care unit or during antenatal consultations of high risk pregnant women. Further studies to explore parental preferences in other cultures and settings are needed to augment our understanding of parental preferences and values.

## Conclusions

In contrast to neonatal practitioners, who make informed, evidence-based decisions for the management of a PDA in preterm infants, mothers who used this particular DAI strongly preferred indomethacin prophylaxis over a symptomatic approach. A shared decision model with family involvement and an in-depth understanding of the potential benefits versus harm of any intervention is in the best interests of the child and should be adopted in clinical practice.

## Additional file

Additional file 1:
**Decision Aid Instrument for Indomethacin Therapy in Preterm Infants.**

## Abbreviations

DAI: Decision aid instrument
ELBW: Extremely low birth weight infant
IVH: Intraventricular hemorrhage
PDA: Patent ductus arteriosus
