# Supplementary material for: A prospective study of maternal preference for indomethacin prophylaxis versus symptomatic treatment of a patent ductus arteriosus in preterm infants
Source: BMC Pediatr. 2015 Apr 22;15:47. doi: 10.1186/s12887-015-0353-4 (PMC4414008; doi:10.1186/s12887-015-0353-4)
Supplement: Additional file 1: — Decision Aid Instrument for Indomethacin Therapy in Preterm Infants. [file 12887_2015_353_MOESM1_ESM.pdf]

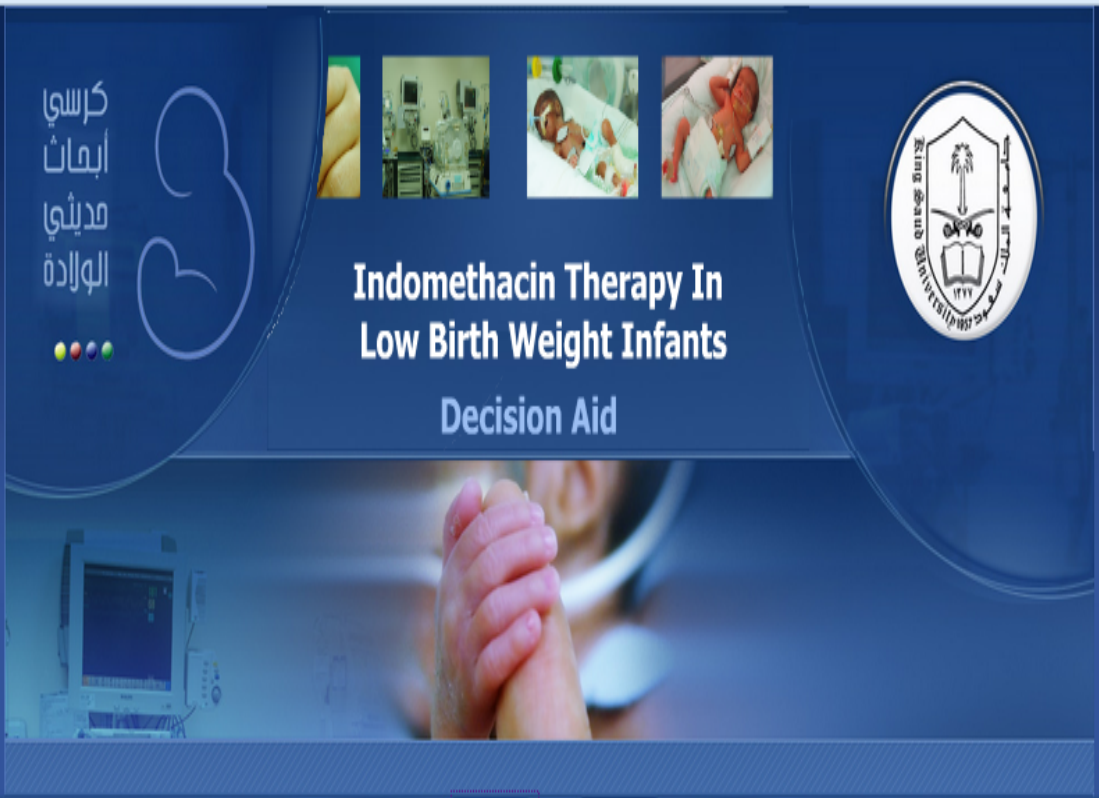

English

عـربي

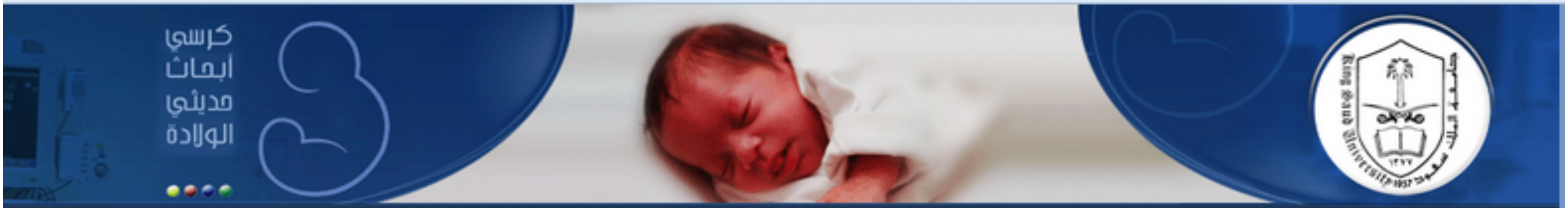

- Introduction
- How this Decision Aid can help you
- Your participation
- How to navigate this site

INTRODUCTION

Indomethacin prophylaxis presents a prophylactic option for preventing patent ductus arteriosis (PDA) in extreme low birth weight (ELBW) infants. Indomethacin prophylaxis has some benefits. But they come with certain downsides. That's why deciding whether to use Indomethacin prophylaxis or not will depend on each individual's values.

If a clinician has told you that Indomethacin prophylaxis is a possible method to prevent PDA in your ELBW infant, this Decision Aid can help you decide whether to use Indomethacin prophylaxis or not.

We believe that your participation in making this treatment decision about your infant's health is very important. However, the degree to which you want to participate is completely up to you.

This decision aid may help you even if you have already made your decision by being a source of information.

Next

جميع الحقوق محفوظة للموقع

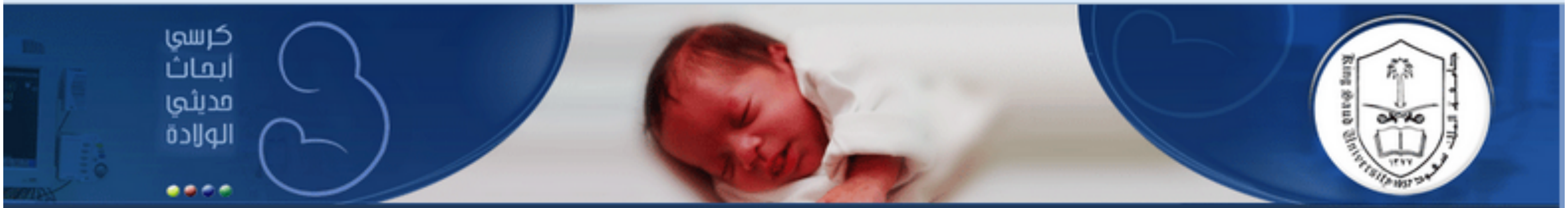

- Introduction
- How this Decision Aid can help you
- Your participation
- How to navigate this site

HOW THIS DECISION AID CAN HELP YOU

The Decision Aid will provide you information about:

- Prematurity and PDA
- Some PDA Related health problems: IVH, BPD
- Indomethacin management options for PDA
- Short term, long term, and side effects related to using Indomethacin including some case scenarios.

The Decision Aid will show you examples how other patients used their values to balance the benefits and downsides and made their decision .

The Decision Aid will then help you with clarifying the values you assign to the different benefits and downsides (your own values and preferences)

Finally, the Decision Aid will help you make your decision by balancing the benefits and risks of the treatment based on your values and preferences .

جميع الحقوق محفوظة للموقع

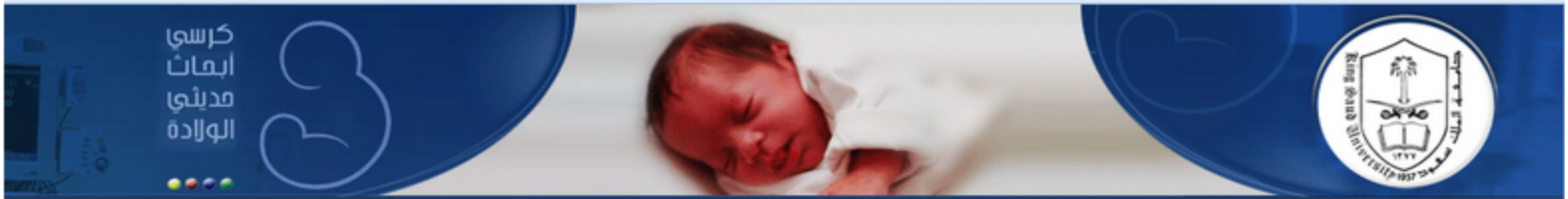

Introduction About Patient Stories Other Patients Your Values Your Decision References Contact Us العربية

- Introduction
- How this Decision Aid can help you
- Your participation
- How to navigate this site

YOUR PARTICIPATION

This Decision Aid allows you to participate to the degree you choose in the decision-making process of using Indomethacin prophylaxis versus symptomatic treatment: There are several approaches:

- You may want to leave the decision entirely up to your care provider.
- You may choose to make the decisions alone.
- You may ask your care provider and/or persons close to you to help you with making the decision.

If you do not care about being involved in making the decision you may choose to quit the Decision Aid at this point and avoid going through other steps. You can then ask your care provider to make this decision for you.

If you do choose to proceed with the Decision Aid, You can then balance the benefits and downsides of using Indomethacin prophylaxis versus symptomatic treatment based on your values. And use this information to make a decision about using Indomethacin prophylaxis versus symptomatic treatment.

If you choose to proceed with the Decision Aid click on the Next Button .

Previous Next

كرسي أبحاث حديثي الولادة

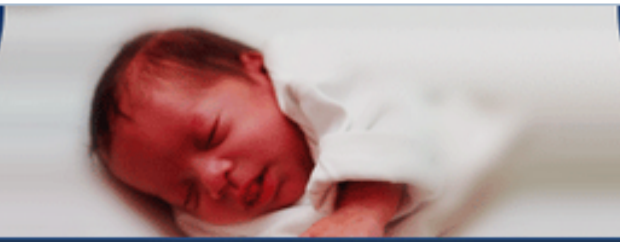

- Introduction
- How this Decision Aid can help you
- Your participation
- How to navigate this site

HOW TO NAVIGATE THIS SITE

To navigate this website you can use a number of navigation buttons:

On the bottom:

- The Back button : Takes you to the previous page
- And Next button : Takes you to the next page

On the top of every page you will find links to the different pages of the Decision Aid. Although you are able to go through these pages in a certain order, the links will allow you to go back and review any previous page.

To start using this Decision Aid click "Next"

جميع الحقوق محفوظة للموقع
تصميم : التكنولوجيا الماسية

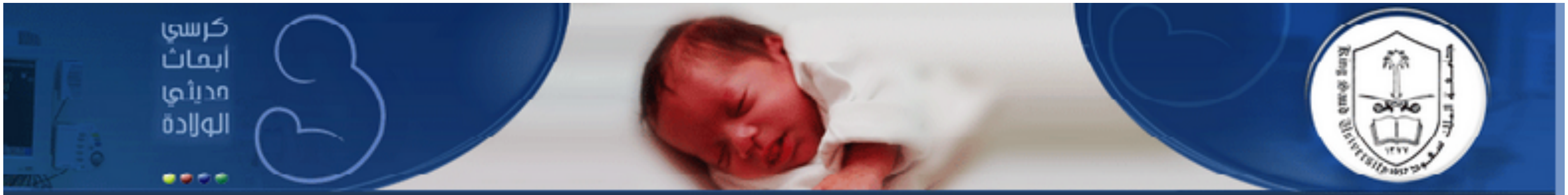

- About prematurity and ELBW
- What is PDA?
- What is IVH?
- What is BPD?
- Management Options

ABOUT PREMATURITY AND ELBW:

Prematurity is defined as babies born before 37 weeks from the first day of the last menstrual period. Very low birth weight (VLBW) babies are those that weigh less than 1.5kg. Some would add a category of extremely low birth weight(ELBW), weighing less than 1.0kg.

A Premature Baby is immature underdeveloped baby, unready for the extra-uterine world due to incomplete development of many of his vital organs such as the lungs and GIT systems, There are many problems and risks associated with a premature baby, such as: PDA, IVH, BPD and breathing problems, body temperature control, feeding problems and others.

Among the risks. We will highlight some important points related to PDA some such as IVH, BPD, and long term outcome, and how Indomethacine can help reduce their risks .

Next

جميع الحقوق محفوظة للموقع

- About prematurity and ELBW
- What is PDA?
- What is IVH?
- What is BPD?
- Management Options

PDA: (Patent Ductus arteriosus)

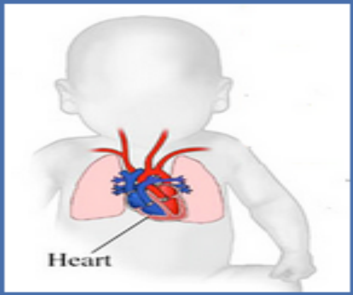

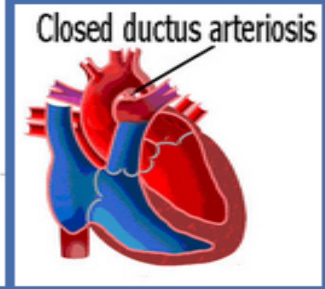

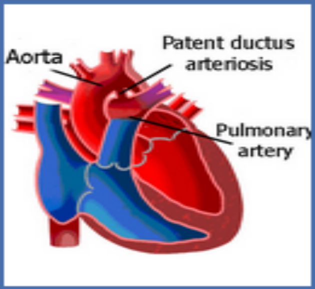

What is Ductus arteriosis?

Ductus arteriosis is a small blood vessel that connects the two major blood vessels coming out of the heart (the pulmonary artery and the aorta) it's essential when the baby is inside the mother's womb to asses in shifting the oxygenated blood from the placenta through pulmonary artery to the aorta and the rest of the baby's body

This duct then closes shortly after delivery when the child starts taking his first breaths and the lung start to take over providing oxygenated blood. However closure could be delayed till few days after delivery and the more premature the baby is, the more delayed the closure will be. In other conditions it could remain patent especially in VLBW infants.

What if it doesn't close?

Patency of this duct a condition called Patent ductus arteriosis, or (PDA) for short, will lead to extra blood flow to the lungs predisposing the child to chronic lung disease or Bronchopulmonary dysplasia or BPD for short, this blood "steal" through the PDA is in the expense of decrease in the blood's flow to other vital organs, such as the brain, intestine and the kidneys,PDA also increases the risk of some conditions such as intraventricular hemorrhage or IVH for short.

Previous Next

جميع الحقوق محفوظة للموقع

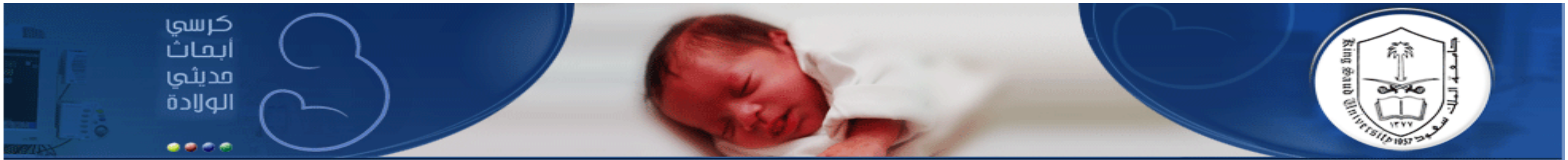

- About prematurity and ELBW
- What is PDA?
- What is IVH?
- What is BPD?
- Management Options

IVH: Intraventricular hemorrhage :

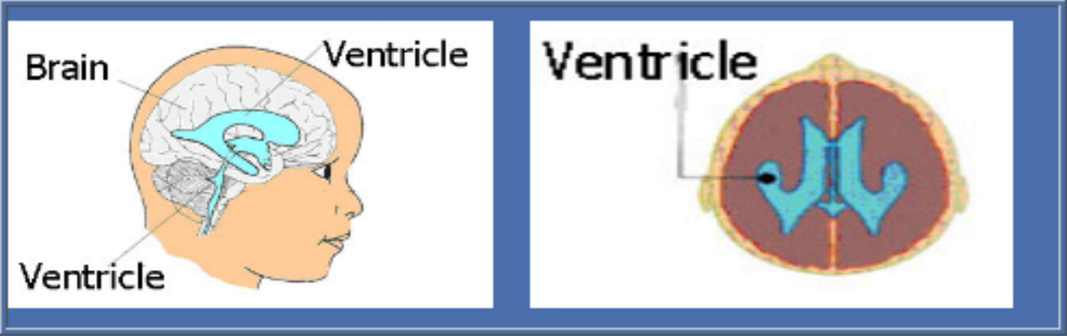

Vessels inside the babies brain are thin and weak, and are sensitive to changes in the blood flow inside these vessels where they can tear easily, fluctuations in blood flow is increased in cases of PDA, this will lead to bruises or intra ventricular hemorrhage (chambers inside the brain filled with a clear fluid where these vessels are), this condition is called "intraventricular hemorrhage" or for short IVH, The bleeding is usually very little, and doesn't cause any symptoms or damage to the brain, but in small proportion of babies the bleeding may be large and cause brain damage or death, IVH is devided into four grades, depending on the extent of hemorrhage, grade I is considered the mildest form with exellent outcome, in contrast with the more sever forms , grade III

and IV where bleeding is associated with dilatation of the ventricles and compression of the brain matter,and in grade IV there is bleeding into the brain matter.

IVH can be detected by Ultra sound device

This is an ultra sound of the brain with white area (pointed by the arrow) representing bleeding within an area in the brain called germinal matrix (rudimentary non functional area): in grade I, IVH

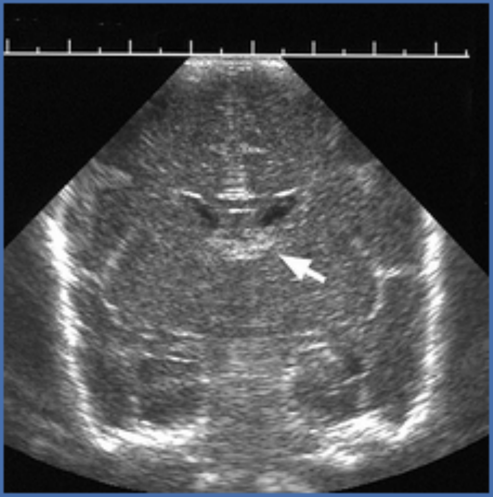

Grade IV: showing bleeding into bilaterally in the periventricular white matter, germinal matrix

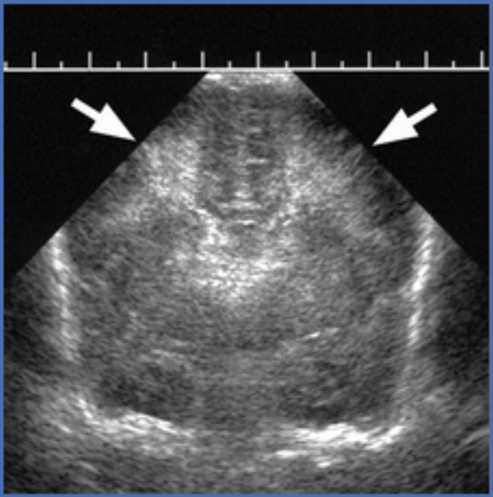

- About prematurity and ELBW
- What is PDA?
- What is IVH?
- What is BPD?
- Management Options

BPD:

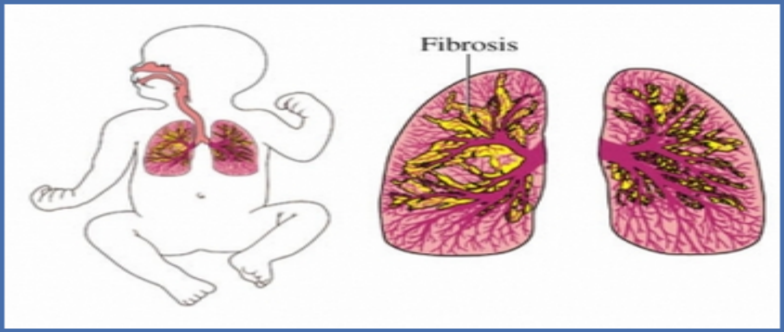

Bronchopulmonary dysplasia (BPD) is a lung injury in preterm infants. PDA increases the risk of BPD due to the overloading of the lung resulting from the ductal patency.

The damaged lung tissue will then be replaced by fibrous tissue, the lung will then not work properlyand babies with BPD will have trouble breathing

The condition usually develops during the first four weeks after birth. Infants then develop increasing oxygen and ventilatory requirements over the first several weeks of life. Many of these patients require supplementary oxygen upon discharge home.

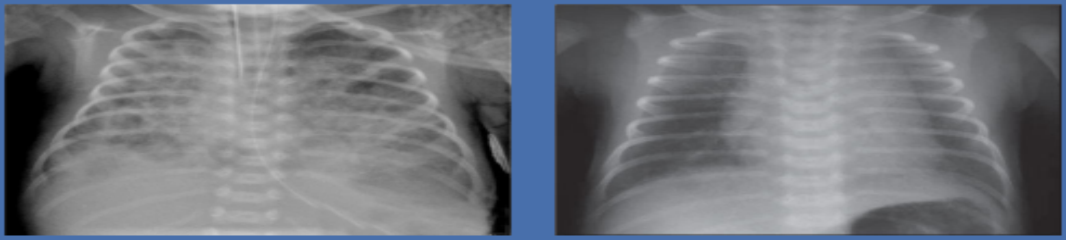

Normal chest x-ray (right) showing clear lung fields in comparison of affected whitish lung fields (left) with BPD

Previous **Next**

جميع الحقوق محفوظة للموقع

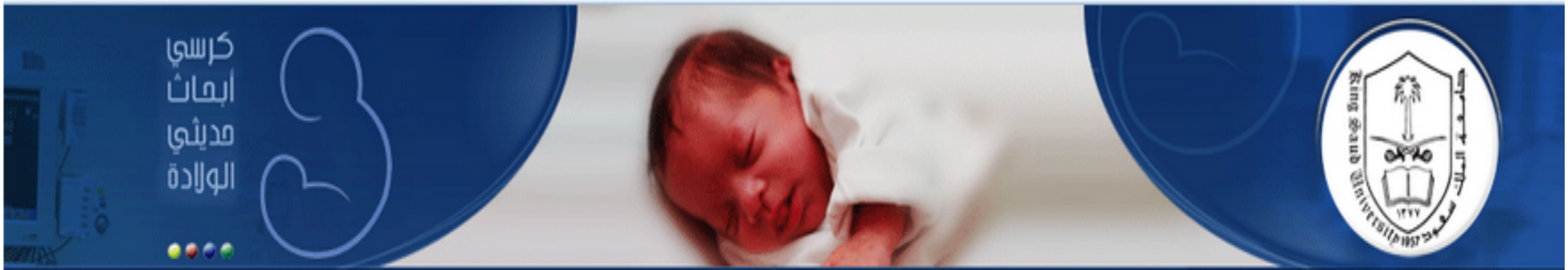

- About prematurity and ELBW
- What is PDA?
- What is IVH?
- What is BPD?
- Management Options

Management options:

Indomethacin: Prophylaxis versus symptomatic treatment:

Indomethacin is a drug that is administered to the premature infant to allow duct closure. There is now a substantial body of literature available to evaluate the role of Indomethacin prophylaxis in the management of the premature infant. Indomethacin prophylaxis reduces the incidence of symptomatic PDA by 55%, severe grade III and IV IVH by 35%, and the need for surgical PDA ligation by 50%, in addition a recent ancillary analysis on the data base of the Trial of Indomethacin Prophylaxis in the Preterm (TIPP trial), showed a significant reduction of serious clinically significant pulmonary hemorrhage during the first week of life. However, this significant reduction in symptomatic PDA, severe IVH, and serious pulmonary hemorrhage did not translate in reduction of mortality, BPD, and more importantly the rates of long term neurosensory outcome .

Treatment Options	Short term outcome	Long term outcome
**Indomethacin Prophylaxis**<br>• Your baby will receive indomethacin through the venous line within the first 6 hours of life for 3 doses given every 12 hours<br>• If your baby develops symptoms of PDA, an ultrasound of the heart will be performed.<br>• If the PDA is still large, he will receive another 3 doses of indomethacin.<br>• If your baby still has large PDA, closure of the PDA by surgery will be considered.	**Out of every 100 premature infants less than 1000 g**<br>• 19 will develop PDA<br>• 6 will require surgery to close the PDA<br>• 9 will develop severe bleed into the brain<br>• 4 will develop sign of damage to the brain (PVL)<br>**Side effects**<br>13 will develop a temporary decrease in urine production	**There is no difference of treatment and prophylaxis options with regard to death and disability rate.**
**Ondomethacin for symptomatic PDA**<br>• Your baby will not receive indomethacin during the first day of his life.<br>• If your baby develops symptoms of PDA (usually in the 3rd day of life), an ultrasound of the heart will be performed. If he/she has a large PDA, indomethacin will be given in 3 doses.<br>• If after the first course, the PDA is till large, another course of indomethacin will be give.<br>• If after the second course, the PDA is still large, closure of the PDA with surgery will be considered.	**Out of every 100 premature infants less than 1000gm**<br>• 43 will develop PDA<br>• 11 will require surgery to close the PDA<br>• 14 will develop severe bleed into the brain<br>• 8 will develop sign of damage to the brain (PVL)<br>**Side effects**<br>7 will develop a temporary decrease in urine production.<br>**We do not know what will happen to your baby!**	

- Baby of Mr. Johns
- Baby of Mr. Smith
- Baby of Mr. Brown

Baby of Mr. Johns:

Baby of Mr. Johns was born at 26 weeks gestation weighing 700 g, he was stabilized in intensive care unit and started on mechanical ventilator and didn't receive Indomethacin prophylaxis, in the 4rth day of life, he started to need more oxygen, and respiratory support, examination revealed an audible murmur (an abnormal extra heart sound), ultra-sound of the heart was done revealed the presence of a 1.5 mm PDA, he was started on Indomethacin treatment for 3 doses, at 0, 12 and 36 hours. Apart from a decrease in urine output during treatment the baby was stable, his symptoms has improved Later on, and heart ECHO repeated showed a closed PDA, his urine output then got normalized .

Next

جميع الحقوق محفوظة للموقع

تصميم : التكنولوجيا الماسية

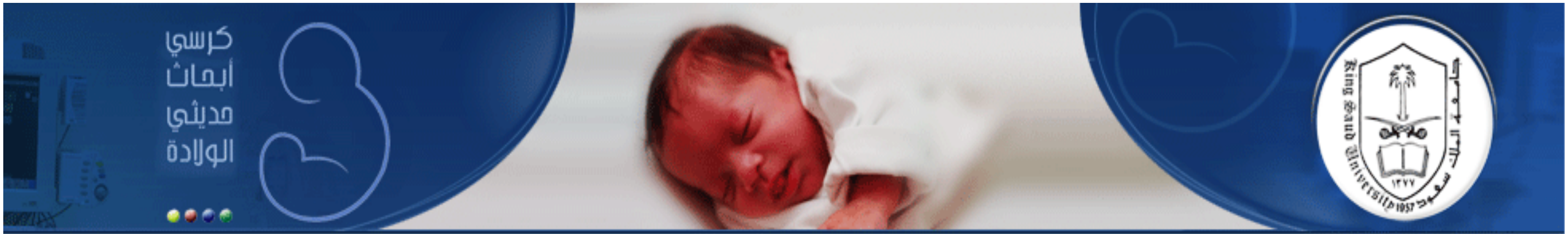

- Baby of Mr. Johns
- Baby of Mr. Smith
- Baby of Mr. Brown

Baby of Mr. Smith:

Baby of Mr. Smith is 28 weeker born with 950 g, in the intensive care unit he was started on mechanical ventilation for respiratory support and he was put on Indomethacin prophylaxis with close monitoring, on the 3rd day where PDA is usually anticipated he was stable, respiratory support settings where successfully decreased, and no clinical evidence of PDA where there.ultrasound of the head showed no intraventricular hemorrhage.

جميع الحقوق محفوظة للموقع
تصميم : التكنولوجيا الماسية

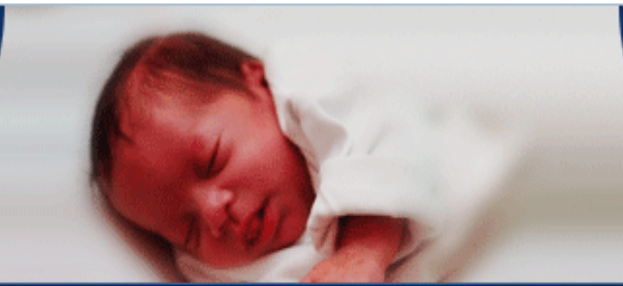

- Baby of Mr. Johns
- Baby of Mr. Smith
- Baby of Mr. Brown

Baby of Mr. Brown:

Baby of Mr. Brown is a preterm born at 24 weeks gestation, he weighs 500g, he was started on Indomethacin prophylaxis and had received 3 doses, on 3rd day clinical evidence of PDA where there, with an audible murmur and general condition deterioration. An ultra-sound of the heart was done, revealed a large size PDA, he was started on a course of Indomethacin treatment of 3 doses given on different time intervals. An ultra-sound was repeated showing persistence of the large PDA, thereafter he got a second Indomethacin course, with no much clinical improvement an ultra-sound was done showing poor response of the PDA to close, baby of Al-Mousa underwent surgical ligation of the PDA, and his symptoms relived and got stabilized after surgery.an ultrasound done on the 5th day of life, shoed grade III IVH.

Previous **Next**

جميع الحقوق محفوظة للموقع
تصميم : التكنولوجيا الماسية

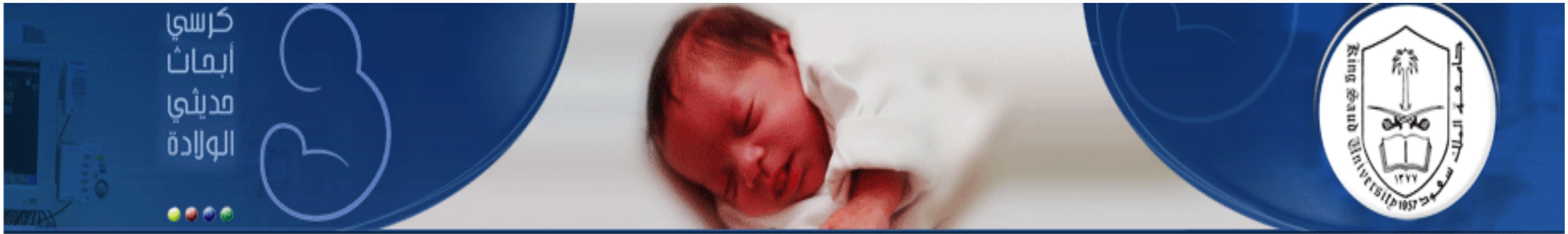

- Mr. Bennett
- Mr. Adams
- Mr.Thomas

Mr. Bennett

Mr. Bennett had a 27 weeker baby with 750 kg and his health care provider informed him that Indomethacin prophylaxis might be an appropriate choice for him. His health care provider asked him to consider the benefits and downsides of using Indomethacin prophylaxis.

He has considered each of these reasons and rated how important they are to him. He indicated the importance of each benefit and downside on a scale from 0 to 100, where 0 indicates the worst imaginable health state (we define it as "dead") and 100 indicates the best imaginable health state.

First Mr. Bennett considered the reasons for using Indomethacin prophylaxis:

- For me, having a PDA in my child is equal to :

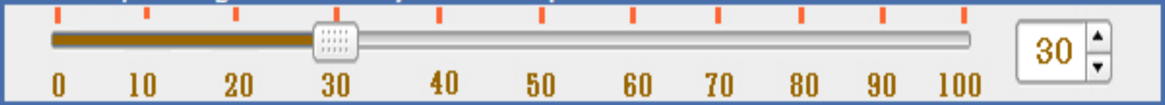

- For me having an IVH in my child is equivalent to:

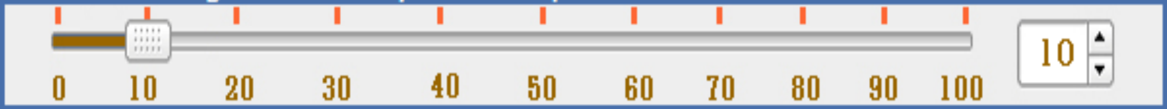

For me having BPD in my child is equal to:

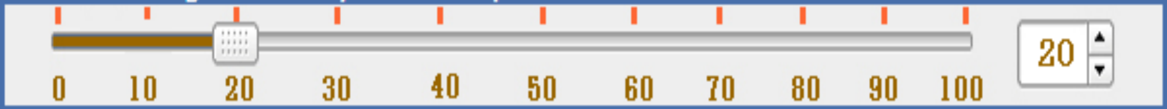

For me having a PDA sever enough to require surgical correction for my child is equal to:

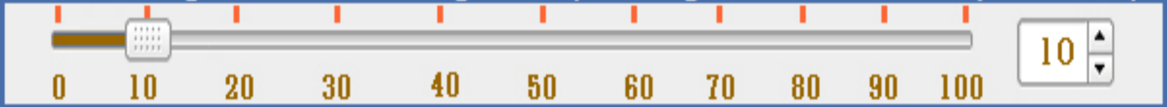

Next, Mr. Bennett considered the reasons for not using Indomethacin prophylaxis

- For me having a temporary decrease in urine output in my child is equivalent to:

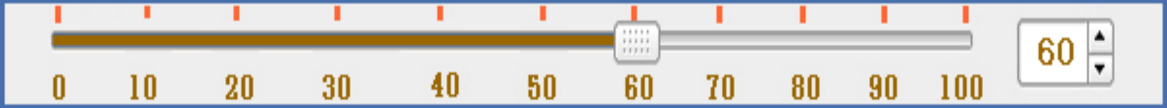

For me getting the fact that there is no effect on my childs neurosensory outcome is equivalent to:

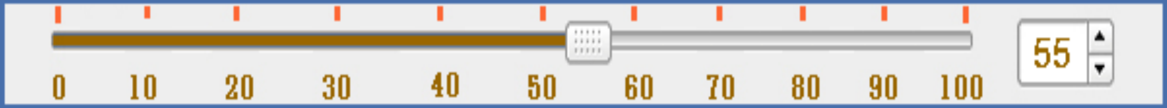

In the next step, Mr. Al-Nasser reviewed a summary of the reasons and their importance to him. He considered this information and his personal feelings to indicate which way he was leaning in his decision.

Mr. Bennett decided to use Idomethacine prophylaxis.

Mr. Bennett's reasons to use Indomethacin:

Indomethacin reduces the chances of developing a PDA
- For me, having a PDA in my child is equal to

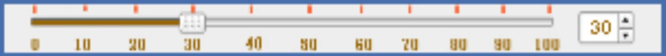

Indomethacin reduces the chances of developing an IVH
- For me having an IVH in my child is equivalent to:

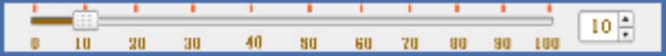

Indomethacin reduces the chances of developing a BOP
-For me having BPD in my child is equal to:

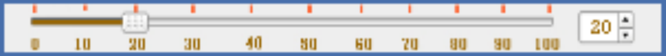

Indomethacin the reduces the chances of a surgical intervention needed for PDA ligation
-For me having a PDA sever enough to require surgical correction for my child is equal to:

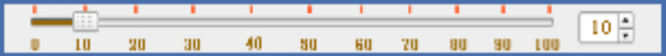

Mr. Bennett's considered the reasons for not using indomethacin prophylaxis:

Indomethacin causes temporary urine outputreduction
- For me having a temporary decrease in urine output in my child is equivalent to:

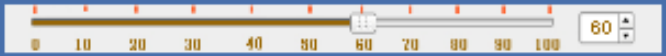

There is no different in my child's neurosensory outcome whether I chosen to commence Indomethacin prophylaxis or not.
- For me getting the fact that there is no effect on my child's neurosensory outcome is equivalent to:

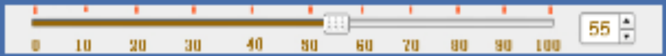

- Mr. Bennett
- Mr. Adams
- Mr.Thomas

Mr. Adams

Mr. Adams had a 26 weeker baby with 700 kg and his health care provider informed him that Indomethacin prophylaxis might be an appropriate choice for him. His health care provider asked him to consider the benefits and downsides of using Indomethacin prophylaxis.

He has considered each of these reasons and rated how important they are to him. He indicated the importance of each benefit and downside on a scale from 0 to 100, where 0 indicates the worst imaginable health state (we define it as "dead") and 100 indicates the best imaginable health state.

First Mr. Adams considered the reasons for using inhaled steroids:

- For me, having a PDA in my child is equal to

0 10 20 30 40 50 60 70 80 90 100 — 10

For me, having an IVH in my child is equal to:

0 10 20 30 40 50 60 70 80 90 100 — 5

For me having a BPD in my child is equal to:

0 10 20 30 40 50 60 70 80 90 100 — 25

For me having a PDA sever enough to require surgical correction for my child is equal to:

0 10 20 30 40 50 60 70 80 90 100 — 10

Next, Mr. Adams considered the reasons for not using Indomethacin prophylaxis

- For me having a temporary decrease in urine output in my child is equivalent to:

0 10 20 30 40 50 60 70 80 90 100 — 90

- For me getting the fact that there is no effect on my child's neurosensory outcome is equivalent to:

0 10 20 30 40 50 60 70 80 90 100 — 50

In the next step, Mr. Adams reviewed a summary of the reasons and their importance to him. He considered this information and his personal feelings to indicate which way he was leaning in his decision.

Mr. Adams decided to use Idomethacine prophylaxis.

Mr. Adams' reasons to use Indomethacin:

Indomethacin reduces the chances of developing a PDA
- For me, having a PDA in my child is equal to

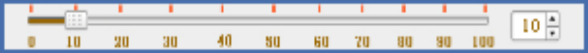

Indomethacin reduces the chances of developing an IVH
- For me having an IVH in my child is equivalent to:

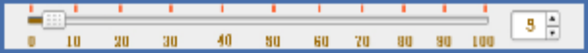

Indomethacin reduces the chances of developing a BOP
For me having BPD in my child is equal to:

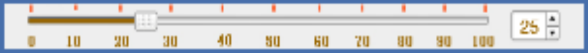

Indomethacin the reduces the chances of a surgical intervention needed for PDA ligation
For me having a PDA sever enough to require surgical correction for my child is equal to:

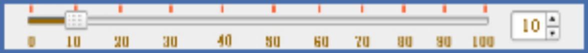

Al-Adams' considered the reasons for not using Indomethacin prophylaxis:

Indomethacin causes temporary urine output reduction
- For me having a temporary decrease in urine output in my child is equivalent to:

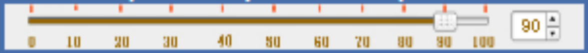

There is no different in my child's neurosensory outcome whether I chosen to commence Indomethacin prophylaxis or not.
- For me getting the fact that there is no effect on my child's neurosensory outcome is equivalent to:

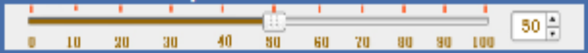

- Mr. Bennett
- Mr. Adams
- Mr.Thomas

Mr. Thomas

Mr. Thomas had a 25 weeker baby with 600 kg and his health care provider informed him that Indomethacin prophylaxis might be an appropriate choice for him. His health care provider asked him to consider the benefits and downsides of using Indomethacin prophylaxis.

He has considered each of these reasons and rated how important they are to him. He indicated the importance of each benefit and downside on a scale from 0 to 100, where 0 indicates the worst imaginable health state (we define it as "dead") and 100 indicates the best imaginable health state.

First Mr. Thomas considered the reasons for using inhaled steroids:

- For me, having a PDA in my child is equal to:

0 10 20 30 40 50 60 70 80 90 100 — 5

For me, having an IVH in my child is equal to:

0 10 20 30 40 50 60 70 80 90 100 — 10

For me having a BPD in my child is equal to:

0 10 20 30 40 50 60 70 80 90 100 — 25

For me having a PDA sever enough to require surgical correction for my child is equal to:

0 10 20 30 40 50 60 70 80 90 100 — 5

Next, Mr. Thomas considered the reasons for not using Indomethacin prophylaxis

- For me having a temporary decrease in urine output in my child is equivalent to:

0 10 20 30 40 50 60 70 80 90 100 — 70

- For me getting the fact that there is no effect on my childs neurosensory outcome is equivalent to:

0 10 20 30 40 50 60 70 80 90 100 — 95

In the next step, Mr. Thomas reviewed a summary of the reasons and their importance to him. He considered this information and his personal feelings to indicate which way he was leaning in his decision.

Mr. Thomas decided to use Idomethacine prophylaxis.

Mr. Thomas' reasons to use Indomethacin:

Indomethacin reduces the chances of developing a PDA

- For me, having a PDA in my child is equal to:

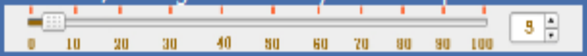

Indomethacin reduces the chances of developing an IVH

- For me having an IVH in my child is equivalent to:

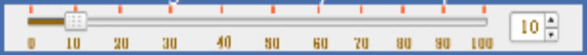

Indomethacin reduces the chances of developing a BOP

-For me having BPD in my child is equal to:

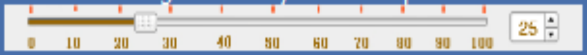

Indomethacin the reduces the chances of a surgical intervention needed for PDA ligation

-For me having a PDA sever enough to require surgical correction for my child is equal to:

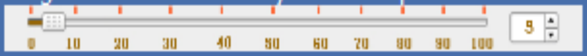

Mr. Thomas' considered the reasons for not using Indomethacin prophylaxis :

Indomethacin causes temporary urine output reduction

- For me having a temporary decrease in urine output in my child is equivalent to:

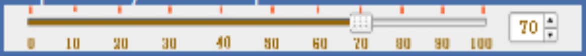

There is no different in my child's neurosensory outcome whether I chosen to commence Indomethacin prophylaxis or not.

- For me getting the fact that there is no effect on my child's neurosensory outcome is equivalent to:

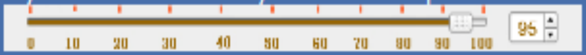

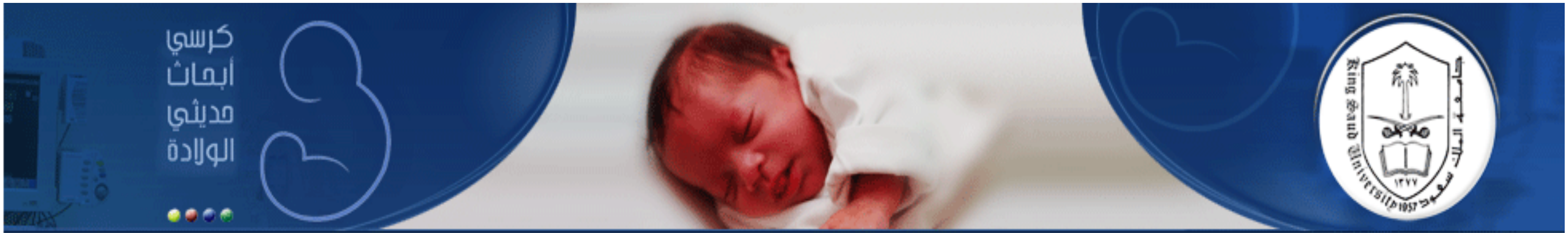

DETERMIMING VALUES

On the following pages we will help you to determine your values related to the benefits and downsides of Indomethacin prophylaxis.

The values you assign will then help balancing benefits and downsides whether Indomethacin prophylaxis vs treatment is right for your child or not, the same way the other parents did. Consider each outcome as it is presented to you, and rate its importance.

You can assign any value between 0 and 100.

0 is equivalent to the worst health state you can imagine (being dead) and 100 to the best health state you can imagine.

Please click on the NEXT BUTTON to continue

Next

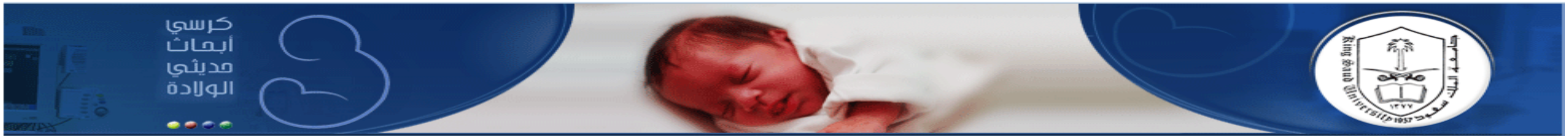

DETERMIMING YOUR VALUES

Please indicate on the scale below how bad or good you think each of the following is by marking on the scale ranging from 0 (dead) to 100 (Full Health). The rating reflects the value you assign to the described option.
Click here for Definition of "Full Health".
Please rate the following:

1- Your child having PDA

50

0 100

2- Your child having IVH

50

0 100

3- Your child developing BPD

50

0 100

4- Your child having a sever enough PDA to undergo surgical correction

50

0 100

5- Your child having a temporary decrease in urine output

50

0 100

6- the fact that there is no difference in long term outcome regarding the neurosensory outcome

50

0 100

Next

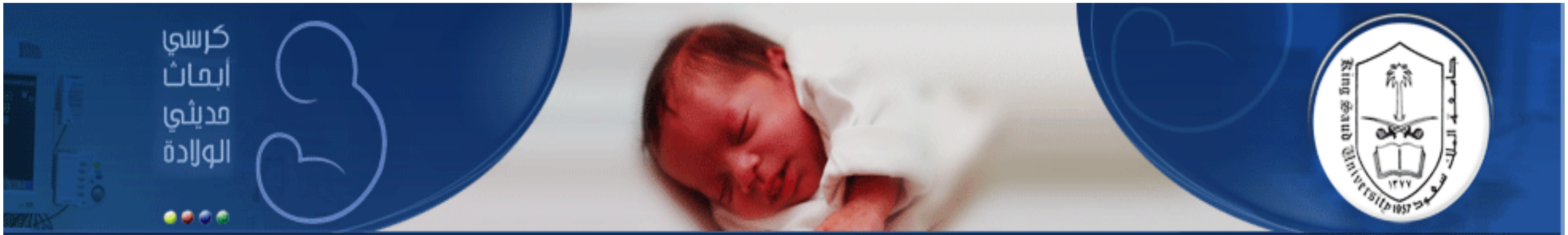

YOUR PARTICIPATION

Now that you have received the medical information, and clarified your values you can choose among the following options:

Balance the benefits and downsides of Indomethacin prophylaxis yourself based on your values. You can then use this information to make a decision about using Indomethacin prophylaxis for your ELBW baby

Your decision:

- I want to use Indomethacin prophylaxis for my child.
- I don't want to use Indomethacin prophylaxis for my child.

جميع الحقوق محفوظة للموقع
تصميم : التكنولوجيا الماسية
